# A Comprehensive Literature Review on Cardioprotective Effects of Bioactive Compounds Present in Fruits of *Aristotelia chilensis* Stuntz (Maqui)

**DOI:** 10.3390/molecules27196147

**Published:** 2022-09-20

**Authors:** Lyanne Rodríguez, Andrés Trostchansky, Hermine Vogel, Irene Wood, Iván Palomo, Sergio Wehinger, Eduardo Fuentes

**Affiliations:** 1 Thrombosis Research Center, Medical Technology School, Department of Clinical Biochemistry and Immunohaematology, Faculty of Health Sciences, Universidad de Talca, Talca 3480094, Chile; 2 Departamento de Bioquímica and Centro de Investigaciones Biomédicas (CEINBIO), Facultad de Medicina, Universidad de la República, Montevideo 11200, Uruguay; 3 Departamento de Horticultura, CENATIV, Facultad de Ciencias Agrarias, Universidad de Talca, Talca 3480094, Chile

**Keywords:** Maqui, platelets, phenolic compounds and cardiovascular

## Abstract

Some fruits and vegetables, rich in bioactive compounds such as polyphenols, flavonoids, and anthocyanins, may inhibit platelet activation pathways and therefore reduce the risk of suffering from CVD when consumed regularly. *Aristotelia chilensis* Stuntz (Maqui) is a shrub or tree native to Chile with outstanding antioxidant activity, associated with its high content in anthocyanins, polyphenols, and flavonoids. Previous studies reveal different pharmacological properties for this berry, but its cardioprotective potential has been little studied. Despite having an abundant composition, and being rich in bioactive products with an antiplatelet role, there are few studies linking this berry with antiplatelet activity. This review summarizes and discusses relevant information on the cardioprotective potential of Maqui, based on its composition of bioactive compounds, mainly as a nutraceutical antiplatelet agent. Articles published between 2000 and 2022 in the following bibliographic databases were selected: PubMed, ScienceDirect, and Google Scholar. Our search revealed that Maqui is a promising cardiovascular target since extracts from this berry have direct effects on the reduction in cardiovascular risk factors (glucose index, obesity, diabetes, among others). Although studies on antiplatelet activity in this fruit are recent, its rich chemical composition clearly shows that the presence of chemical compounds (anthocyanins, flavonoids, phenolic acids, among others) with high antiplatelet potential can provide this berry with antiplatelet properties. These bioactive compounds have antiplatelet effects with multiple targets in the platelet, particularly, they have been related to the inhibition of thromboxane, thrombin, ADP, and GPVI receptors, or through the pathways by which these receptors stimulate platelet aggregation. Detailed studies are needed to clarify this gap in the literature, as well as to specifically evaluate the mechanism of action of Maqui extracts, due to the presence of phenolic compounds.

## 1. Introduction

Cardiovascular diseases (CVDs: acute myocardial infarction, cerebrovascular disease, and peripheral arterial thrombosis) are responsible for approximately 30% of deaths worldwide [1,2]. Platelets play a relevant role in the atherosclerotic process in physiopathologic and thrombotic events. It has been observed that some bioactive compounds present in fruits and vegetables, when consumed regularly, can inhibit platelet aggregation and thus reduce the risk of CVD [3].

Epidemiological studies have shown that modifiable cardiovascular risk factors (CVRFs) increase the probability of suffering from CVD [4]. There are non-modifiable factors such as age and genetic predisposition in addition to modifiable CVRFs such as smoking, dyslipidemias, hypertension, diabetes, metabolic syndrome, and overweight/obesity [5,6,7]. It has been previously described how inflammation and thrombosis are involved in the onset and progression of non-communicable diseases [8].

Modifications in the population’s lifestyle have effects on CVD. Consuming a healthy diet, rich in fruit and vegetables, with high concentrations of proven bioactive protective and antioxidant compounds, has shown to be a promising action in the prevention of CVD [9,10]. Diet and lifestyle are modifiable risk factors that can have a significant impact on an individual’s likelihood of developing non-communicable diseases. It has become clear that a person’s nutritional status is an important factor in preparing the immune system to deal with any disease [8]. 

The ethnomedicinal use of natural products and their natural bioactive compounds has increased for the treatment and prevention of CVD [11]. The traditional Mediterranean diet, as well as medicinal plants, have also been reported to exert cardioprotective and antiplatelet effects in the primary and secondary prevention of CVD [12]. According to estimates by the World Health Organization (WHO), approximately 80% of the world’s population uses traditional medicinal herbs for their primary health care. Additionally, the consumption of berries, for example, chokeberries (*Aronia melanocarpa*), blueberries (*Vaccinium sect. Oxycoccus*), sea buckthorn berries (*Hippophae rhamnoides*), and grapes (*Vitis*), as well as their various derivative commercial drugs, has been linked to the prevention of CVD, such as atherosclerosis in elderly men [13].

Foods, fruits, vegetables, grains, and fermented beverages such as wine and beer, with antithrombotic and anti-inflammatory properties, contain a large number of phytochemicals such as phenolic compounds, carotenes, alkaloids, terpenes, peptides, and bioactive lipid molecules. Many of these compounds exhibit potent inhibition or modulation of several key proinflammatory and prothrombotic mediator signaling pathways, such as platelet-activating factor (PAF), thrombin, collagen, ADP, arachidonic acid, and related eicosanoids [8]. It has been described that the modulation of the intracellular oxidative state through the consumption of antioxidants in the diet could be a promising approach to reducing the risk of CVD [14].

Polyphenols are distinct in the Mediterranean diet because they can modulate platelet function through different mechanisms of action, e.g., modulating thromboxane formation. It is well known that phenolic compounds are the main components of many plants, and have gained increasing public and scientific interest due to their beneficial effects on health as antioxidants [14]. Therefore, plants with high polyphenol content and antioxidant activity are worth studying. In this group of plants of great interest, we have *Aristotelia chilensis* (Stuntz) also known as Maqui, which is a plant native to Chile, a member of the family Eleocarpaceae, which is distributed across the world in tropical and temperate areas of Asia, Oceania, and South America. Maqui has stood out for the presence of phenolic compounds and its anticancer, antimutagenic, anti-inflammatory, and antioxidant activities. Although some authors have discussed Maqui’s cardioprotective activity, there exist few studies linking this berry with an antiplatelet effect.

### Maqui: Relevance and Traditional Uses

Maqui is a sacred medicinal plant to the indigenous Mapuche people, native to Chile and the Argentinian border [15]. Its light to dark purple berries ripen between December and February [16,17,18]. Besides its medicinal use, Maqui is consumed and prepared as food (juice, pulp, jam) or liquor [19]. Maqui has a high phenol content and, depending on the solvent used for its extraction, the phenolic content of the fruit can reach up to 51 g GAE/kg [16,19]. Aqueous and ethanolic extracts of Maqui fruit have been used since ancient times for medicinal purposes to treat digestive disorders, inflammation, and migraines, while leaf extracts exert antiseptic, anti-inflammatory, and indigestion protective properties [20]. Most of the actions described for this berry are related to the high content of anthocyanins and polyphenols in the ripe fruit [21,22]. 

Many collectors and businessmen have opted for plantations of this native fruit, which is mainly exported to Europe and Asia. Around 170,000 hectares are planted throughout Chile (estimate of the area of wild Maqui indicated by collectors in the agricultural census), but the growing demand for Maqui is not satisfied by wild production. In recent years, the demand from the food and pharmaceutical industry for Maqui berries has increased, which prompted the domestication of the species to achieve greater availability and avoid the destruction of wild populations [23]. Studies of the genetic structure of natural populations were carried out, looking for genotypes to guide the selection of clones in addition to establishing the best agronomic parameters for their selection and cultivation [24,25]. For this, studies of the genetic diversity of different Maqui populations using molecular marker techniques such as chloroplast microsatellites and amplified fragment length polymorphisms (AFLPs) guided the domestication of this species [23,24]. More than 60 clones of Maqui trees were planted at the Experimental Station of the University of Talca (Panguilemo) and genetic variability was evaluated using molecular markers [24]. The most outstanding clones were planted in other experimental stations (Los Niches, Chillan, and Río Negro), which allowed a more detailed evaluation of them [23,26]. From these works, the clones Luna Nueva, Morena, and Perla Negra present relevant agronomic characteristics (harvest yield, fruit size, and early maturation) [24,27], making them suitable for further nutraceutical evaluations. 

In wild plantations, about 50% of the fruit is immature at harvest, while in the domesticated tree this is only 20% [27]. Currently, only the ripe fruit is used in the food and pharmaceutical industry [19,28], while the immature fruit has no application and is discarded. Likewise, Maqui leaves constitute agroindustrial waste and contain large amounts of total phenols [29]. 

The genetic differences in Maqui clones significantly influence their biological potential [24]. Although the biological properties of Maqui have been extensively studied, few reports analyze its antiplatelet activity. However, when evaluating Maqui’s composition, some compounds present in this berry have been shown to exert inhibition of platelet activation, secretion, and aggregation, thus reducing the risk of CVD [30,31].

In the current review, we will discuss and summarize the studies that relate Maqui with cardiovascular protection, specifically in the prevention of CVRFs. On the other hand, we will discuss how Maqui’s bioactive compounds mainly present in its fruit modulate platelet function as a mechanism of CVD protection.

## 2. Platelets and Atherothrombosis

Platelets are anucleated cells (1.5–3.0 μm in diameter) that originate from the fragmentation of the cytoplasm of the megakaryocyte through endomitosis. This process leads to the formation of platelets that enter the bloodstream [32,33]. 

Platelets circulate as discoid-shaped elements. In response to vascular damage, they emit pseudopodia, secreting the content of their granules and remodeling their membrane [32]. They are formed by a plasma membrane expressing important glycoproteic receptors: collagen receptors (glycoprotein (GP) IIb/IIa, GPIb/IX, GPVI, GPIa/IIa, GPIV), and non-glycoproteic receptors: adenosine diphosphate (ADP) receptors (P2Y1, P2Y12), thrombin receptors (PAR1, PAR4), thromboxane A2 receptor (TxA2), serotonin receptor, and prostacyclin I_2_ receptor. The receptor–agonist interaction participates in the outside/inside or inside/outside platelet signaling leading to platelet activation and/or inhibition [32,34]. 

**Figure 1:**
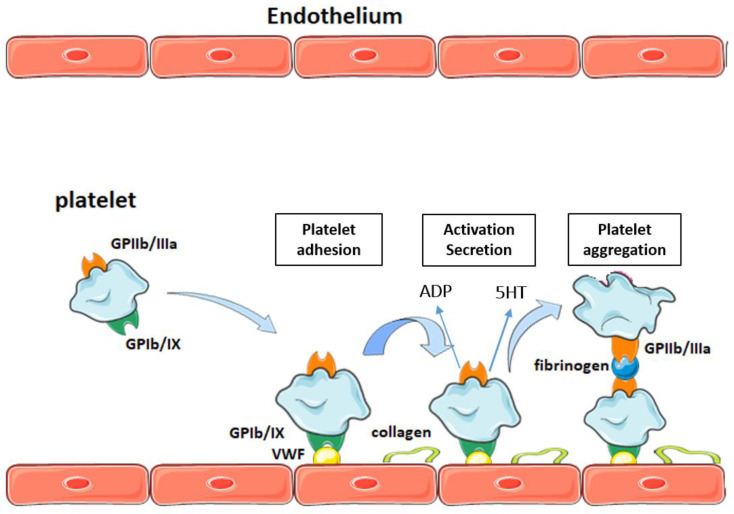
Platelet function in the process of primary hemostasis. ADP, adenosine diphosphate; VWF, von Willebrand factor; GP, glycoprotein; 5HT: serotonin.

Platelets, in their functional aspects, involve the processes of adhesion, activation, secretion, and platelet aggregation (Figure 1). Adhesion occurs in response to vascular damage; platelet membrane receptors interact with their respective ligands and bind to the injured wall resulting in platelets’ morphological changes and secretion of their alpha and dense granule content [35]. Then, platelets begin the aggregation process which is characterized by platelet–platelet binding to form a platelet aggregate. Their functionality can be modified by CVRFs, e.g., developing a platelet hyperactivation state [36]. This condition has been described as an increase in the capacity of activation, secretion, and aggregation against low concentrations of agonists, due to alterations in different signaling pathways [37,38].

### Participation of Platelets in Atherothrombosis

**Figure 2:**
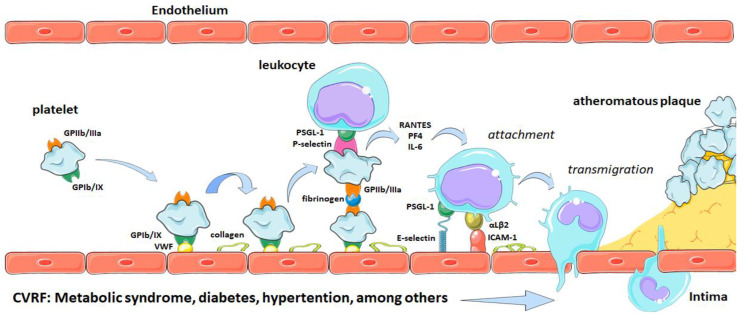
Participation of platelets in the atherothrombotic process. VWF, von Willebrand factor; GP, glycoprotein; IL, interleukin, PF4, platelet factor 4; PSGL-1, glycoprotein ligand-1 P-selectin; RANTES, beta-regulatory chemokine; VCAM-1, vascular cell-1 adhesion molecule.

Platelets represent the bridge between an inflammatory process and thrombosis, a fundamental process for the development of atherothrombosis and complication of atherosclerotic plaque [39]. Plaque rupture leads to the formation of a thrombus rich in platelets, a process characteristic of arterial thrombosis (Figure 2). During the atherothrombotic process, platelets participate in both the initial and final stages [40]. In the initial phase, platelets adhere to the CVRF-damaged endothelium, secreting and exposing molecules that amplify the inflammatory process [35,40]. In the final stage, after the plaque ruptures, the platelets adhere at endothelium formatting to aggregates and thus contribute significantly to thrombus formation [41].

It has been observed that some bioactive compounds in fruits and vegetables, polyphenols and flavonoids, when consumed regularly may inhibit platelet activation and therefore reduce the risk of CVD. The importance of natural antioxidants to provide cardiovascular protection has been highlighted before [3,42,43], as well as the reports about the antithrombotic activity of fruits and vegetables [44,45]. In the case of Maqui, these studies are scarce [20,30]. 

## 3. Chemical Characterization of Maqui

The development of chromatographic and spectrophotometric techniques such as high-performance liquid chromatography (HPLC), coupled with diode detectors (DADs) and a mass spectrometer (MS) have favored advances in the identification and quantification of anthocyanins and other polyphenolic compounds [46,47]. 

**Figure 3:**
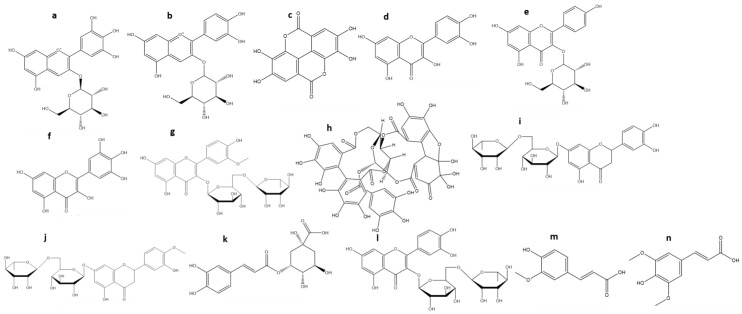
The chemical structure of the main chemical compounds present in the fruits of Maqui. Chemical structures corresponding to **a**, Delphinidin-3-glucoside; **b**, Cyanidin-3-glucoside; **c**, Ellagic acid; **d**, Quercetin; **e**, Kaempferol-3-glucoside; **f**, Myricetin; **g**, Isorhamnetin-3 -rutinoside; **h**, Granatin B; **i**, Eriodic-tyol-7-rutinoside; **j**, Hesperetin-7-rutinoside; **k**, 5-*O*-caffeoylquinic acid; **l**, Rutin; **m**, Ferulic acid and **n**, Sinapic acid.

The main bioactive compounds already reported in Maqui include phenolic acids (caffeic and gallic), flavonols (quercetin, rutin, and myricetin), flavonoids (catechin and epicatechin), and anthocyanins (delphinidin and its derivatives, malvidin, petunidin, cyanidin, and peonidin) [18] (Figure 3). 

Aqueous extracts of the fruits of Maqui contain a high content of phenolic acids (ferulic acid, gallic acid, caffeic acid), flavonoids (quercetin, myricetin, kaempferol, delphinidin, and cyanidin) and tannins (ellagitannins) [48]. On the other hand, the hydroalcoholic extract of the Maqui fruit presents high values of phenols and the presence of hydrophilic compounds, coumaric, gentisic, ferulic, and gallic acids, delphinidin-3,5-*O*-diglucoside, cyanidin-3-*O*-glucoside, and proanthocyanidin B, as well as hydrophobic compounds, catechins, quercetin, rutin, and anthocyanidins [49,50,51]. Cespedes et al. evaluated an ethanolic extract by extracting first with EtOH/H_2_O (6:4) followed by a subsequent extraction with H_2_O. This extract presented a composition based on gentisic acid, ferulic acid, gallic acid, p-coumaric acid, sinapic acid, 4-hydroxybenzoic acid, delphinidin, cyanidin, vanillic acid, delphinidin gallate, gallocatechin gallate, quercetin, rutin, myricetin, catechin and epicatechin, and anthocyanin glycosides [52]. Another study indicates the phytochemical characterization of several Maqui berries by HPLC-DAD-ESI/MS^n^. Eight glycosylated anthocyanins, derived from cyanine and delphinine, have been reported: delphinidin-3-*O*-sambubioside-5-*O*-glucoside, delphinidin-3,5-*O*-diglucoside, cyanidin-3,5-*O*-diglucoside, cyanidin-3-*O*-sambubioside-5-*O*-glucoside [53]. Sonication has been reported as an ideal alternative to obtain extracts from Maqui berries with high bioactivity, increasing the content of anthocyanins in Maqui berries, 3-glucosides, 3,5-*O*-diglucosides, 3-*O*-sambubiosides, and 3-*O*-sambubioside-5-*O*-glucosides of delphinidin and cyanidin as determined by HPLC with photodiode array and MS detection [46]. Maqui fruit extracts’ main components are summarized in Table 1.

Only a few studies refer to the phytochemical analysis of leaf extracts of Maqui. The presence of indole and quinoline alkaloids has been reported in the leaves [19], being identified as aristoteline, serratoline, aristone, horbatinol, and horbatina [54,55]. Studies performed on ethanolic extracts of Maqui leaves reported the presence of some polyphenols such as gallic acid, coumaric acid, quercetin, myricetin, rutin, pelargonidin, and catechin. Interestingly, these compounds have been associated with the prevention of some CVD [56].

A study carried out in different regions of Chile showed that the total anthocyanin concentrations (TA) vary between 660 and 1500 mg cyanidin-3-*O*-glucoside/100 g of dried fruit, while the TP ranges between 1070 and 2050 mg GAE/100 g of dried fruit [17]. The total content of anthocyanins and polyphenols in Maqui is highly variable, depending on growing conditions, harvest times, plant genotype [17], and the different extraction procedures [57]. Maqui fruits have higher total polyphenol (TP) levels and antioxidant activity than other species recognized for their high phenolic content such as blueberries, pomegranates, blackberries, and red raspberries [58,59]. If we compare the Maqui with other berries, for example, Euterpe oleracea, better known as açai, which has also stood out for being one of the most nutritious fruits in South America, the phenolic content does not exceed the values referenced for Maqui [60]. 

**Table 1:** Anthocyanins and phenolic compounds identified and quantified in Maqui fruit extracts.

Anthocyanins (TA)	% Average (Range)	References
Delphinidin-3-*O*-sambubioside-5-*O*-glucoside	32.4 (15–49)	[21,46,52,53,61]
Delphinidin-3-*O*-glucoside (**a**)	18.6 (11–28)	[21,46,52,53,61]
Delphinidin-3,5-*O*-diglucoside	18.3 (14–24)	[46,52,53,61]
Delphinidin-3-*O*-sambubioside	10 (6–16)	[21,46,52,53,61]
Cyanidin-3-*O*-glucoside (**b**)	11 (6–16)	[46,52,61]
Cyanidin3,5-diglucoside	10.8 (7–14)	[21,46,52,53]
Cyanidin-3-*O*-sambubioside-5-*O*-glucoside	9 (7–11)	[52,61]
Cyanidin-3-*O*-sambubioside	6.5 (6–7)	[21,46,52,53]
Cyanidin-*O*-glucoside-5-*O*-rhamnoside	1.5 (1–2)	[21,53]
Phenolic Compounds (TP)		
Ellagic acid (**c**)	30	[22]
Ellagic acid rhamnoside	8	[62]
Ellagic acid hexoside	2.8 (2–3.5)	[22,53,62]
Quercetin-*O*-galloyl-*O*-hexoside	24	[22]
Quercetin-3-*O*-rutinoside	10 (7–13)	[53,62]
Quercetin-3-*O*-arabinoside	6 (5–7)	[22,53,62]
Quercetin-3-*O*-galactoside	6 (5–7)	[22,53,62]
Quercetin-3-*O*-rhamnoside	5.5 (4.1–6)	[53,62]
Quercetin-3-*O*-xyloside	3.5 (2–5)	[22,53,62]
Quercetin-3-*O*-glucoside	3 (2–4)	[22,53,62]
Quercetin (**d**)	2	[22]
Kaempferol-3-*O*-glucoside (**e**)	18	[53]
Kaempferol-3-*O*-galactoside	12	[53]
Kaempferol-3-*O*-rutinoside	2	[53]
Myricetin-3-*O*-glucoside	13 (6–20)	[22,53]
Myricetin (**f**)	8	[22]
Myricetin-3-*O*-galactoside	6.3 (4–10)	[22,53,62]
Myricetin-3-*O*-galoyl-*O*-glucoside	4 (2–6)	[22,53,62]
Myricetin-3-*O*-galoyl-*O*-glucoside	4 (2–6)	[22,53,62]
Isorhamnetin-3-*O*-rutinoside (**g**)	2	[53]
Granatin B (**h**)	20	[62]
Eriodictyol-7-*O*-rutinoside (**i**)	11	[62]
Hesperetin-7-*O*-rutinoside (**j**)	11	[62]
5-*O*-caffeoylquinic acid (**k**)	8.5 (5–12)	[22,53,62]
Rutin (**l**)	6	[22]
Ferulic acid (**m**)	4	[62]
Sinapic acid (**n**)	3	[62]

Average % (range): percentage in which the compound is present in the Maqui fruit.

## 4. Cardioprotective Role of Maqui 

**Figure 4:**
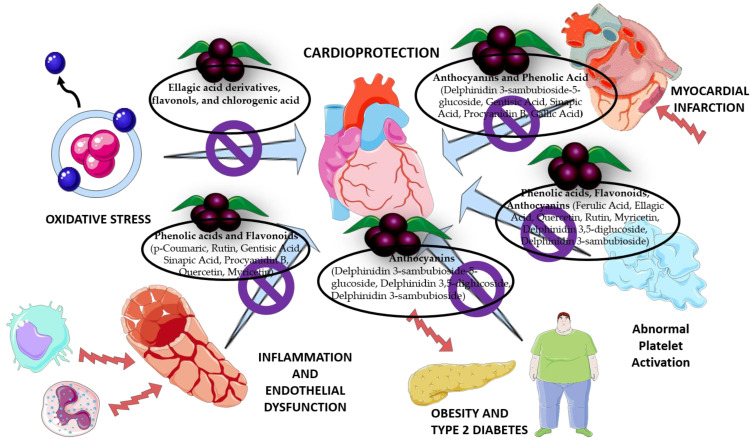
Cardioprotective effect of chemical compounds identified in Maqui.

Food and/or pharmaceutical supplements have become attractive alternatives to reducing CVRFs [63]. Some non-communicable diseases, including CVD, could be prevented by improving the lifestyle of the population, including the consumption of a healthy diet [64]. Studies have highlighted the importance of natural antioxidants present in vegetables, to provide cardiovascular protection [42]. An investigation showed the cardioprotective effect of proanthocyanidins present in a grape seed extract evaluated in mice [65]. Some works refer to the antioxidant activity of the methanolic extract of the fruit of Maqui and the cardioprotective effects on acute ischemia/reperfusion induced in the hearts of rats [20]. It has been described that a diet rich in fruits and vegetables favors adequate platelet function, with positive effects on cardiovascular health [30]. Undoubtedly, the Maqui compared to other fruits presents a wide biological potential, being a promising target for study mainly in the cardiovascular area (Figure 4).

### 4.1. Antioxidant Effect

Investigations highlight the antioxidant potential of Maqui, which is more potent compared to other berries; the antioxidant capacity expressed as Trolox equivalents is higher in Maqui berries compared to *Vaccinium floribundum* [61]. Other authors also point out that this berry has a better potential for radical scavenging and antioxidant reactivity using in vitro antioxidant capacity tests [66]. Some works refer to the antioxidant activity of the methanolic extract of the Maqui fruit, and the cardioprotective effects on acute ischemia/reperfusion induced in an in vivo rat model. In this study, the methanolic extract protected the animals from cardiac damage due to the incidence of reperfusion dysrhythmias and non-recovery of sinus rhythm. The antioxidant effect of this extract was also evaluated with ORAC, FRAP, and DPPH assays presenting an IC_50_ of 1.62 ppm against DPPH [20].

It has been shown that the antioxidant activity of the Maqui berry is directly related to cognitive protection and a decrease in oxidative stress markers. Methanol/water extracts and their partitions (acetone and ethyl acetate) of three Maqui varieties were studied for antitumor effects in HT-29, Caco-2, and NF-κ colon cancer cells. The inhibition of cell growth and nitric oxide (NO) production by the most active extracts was dose-dependent and significant effects were observed at concentrations of 25.0 and 10.0 ppm, respectively [67]. 

Maqui berries have shown better results against different in vitro tests of antioxidant activity [68]. A new drink based on lemon juice and Maqui has been compared against other mixtures with açai and blackthorn. The Maqui mixture was the most interesting in terms of antioxidant capacity. An ABTS assay showed that Maqui-containing mixtures presented better results (8.35 ± 0.55 mM Trolox), supporting the strong antioxidant activity of this berry. Açai blends showed lower capacity compared to Maqui blends (3.88 ± 0.58 mM Trolox). This study also showed the most potent reducing capacity of Maqui compared to açai against the DPPH radical (3.07 mM ± 0.09 mM Trolox and 0.12 ± 0.19 mM Trolox, respectively) [69]. 

### 4.2. Effect on Inflammation and Endothelial Dysfunction

Maqui has received attention due to its broad biological potential, including reducing inflammation. In vitro studies have mainly shown that Maqui limits adipogenesis and inflammatory pathways [61] in addition to affecting the production of NO [70]. A hydroalcoholic extract of this species, as well as rutin, improved endothelium-dependent relaxation and reduced plasma levels of cholesterol, LDL, and triglycerides. Additionally, Maqui and rutin improve the bioavailability of NO [71]. Another study evidenced the protective effect of a hydroethanolic extract of Maqui and its flavonoids (rutin and quercetin) on endothelial dysfunction induced by high glucose and pyrogallol, through a greater generation and bioavailability of NO [72]. An aqueous extract of Maqui berries modulated NO production in a hyperglycemic and/or hyperinsulinemic microenvironment, in addition to improving endothelial function, through the possible vasorelaxant properties of NO [70]. Maqui methanolic extracts under inflammatory conditions suppress NO production, through the regulation of inducible nitric oxide synthases (iNOSs). These results correlate with the inhibition of the expressions of NF-κB and COX-2 proteins [67]. On the other hand, the methanolic extract of the Maqui fruit decreases lipid oxidation and reduces the concentration of thiobarbituric acid reactive species (lipid peroxidation index) [20]. In human endothelial cell cultures, Maqui juice dose-dependently protects intracellular oxidative stress induced by hydrogen peroxide, suggesting that it might have antiatherogenic properties [66].

### 4.3. Effect on Diabetes and Obesity

Maqui dietary supplementation has been shown to have positive effects on fasting glucose and insulin levels in human and mouse models of type 2 diabetes and obesity. Maqui standardized extract showed a significant reduction in glycemia in 10 volunteers with moderate glucose intolerance [73]. It was shown that obese mice induced by a high-fat diet and supplemented with lyophilized Maqui had better insulin response capacity, less weight gain, and greater thermogenic activity [74].

The effect of Maqui anthocyanins on a murine model of type 2 diabetes was studied. Oral administration of these anthocyanins improved fasting blood glucose levels and glucose tolerance in hyperglycemic obese C57BL/6J mice, fed a high-fat diet. On the other hand, it also improved the negative regulation of the gluconeogenic enzyme glucose-6-phosphatase stimulated by insulin and decreased glucose production. Additionally, delphinidin-3-*O*-sambubioside-5-*O*-glucoside was shown to dose-dependently lower fasting blood glucose levels in obese C57BL/6J mice [75]. 

Maqui berries are commercially extracted to produce a standardized polyphenolic extract, which contains 25% delphinidin, the most abundant anthocyanin in this fruit. The extract has other constituents, phenolic acids, flavonols (quercetin, rutin, myricetin, and flavanols (catechins and epicatechins). Maqui berry extract is marketed worldwide as Delphinol^®^ and is distributed by the company Maqui New Life (MNL, trademark owner), based in Santiago, Chile, and Cham, Switzerland [76]. Delphinol^®^ is a nutritional supplement with the ability to naturally control postprandial glycemia through the inhibition of the sodium and glucose cotransporter in the small intestine. An investigation carried out on ten volunteers with moderate glucose intolerance, using a double-blind, placebo-controlled, crossover study model, showed the effect of Delphinol^®^ on glucose. This supplement significantly inhibited non-desired changes in postprandial blood glucose levels, 60 and 90 min after ingestion of boiled rice [73]. Alvarado et al. showed that the effects of Delphinol^®^ on basal glycemia and insulinemia could be related to the inhibition of intestinal glucose transporters, as well as an incretin-mediated effect or by improving sensitivity to insulin [77].

Additionally, the role of Maqui extracts has been highlighted not only in preventing diabetes but also in preventing and treating obesity. A high-fat diet supplemented with a regular dose of Maqui berries shows better insulin response and less weight gain. In addition, a differential expression of genes involved in de novo lipogenesis, fatty acid oxidation, the formation of multilocular lipid droplets, and thermogenesis in subcutaneous white adipose tissue (scWAT) is evidenced. These findings highlighted the role of this berry in preventing or treating type 2 diabetes and obesity-related diseases, as well as their metabolic complications [74].

Alpha-glucosidase inhibition is considered one of the measures to regulate type 2 diabetes [69]. Gironés-Vilaplana et al. (2014) reported that EC_50_ values of glucosidase inhibition for açai and Maqui berries were in the range of 0.33–2.14 mg/mL, while for strawberry pomace the values were not so influential, which shows once again the potential of Maqui over other berries [69,78]. Maqui has shown potent postprandial glycemic lowering effects at a single dose of approximately 1000 µmol GAE of polyphenols derived from Maqui berry extract and lemon juice, following carbohydrate (glucose and rice) ingestion [79]. A similar dose was also effective in lowering postprandial blood glucose (induced by ingestion of 25 g glucose) by ingestion of coffee containing 2.5 mmol/L chlorogenic acid (a total amount of 1000 μmol chlorogenic acid) [79,80]. Table 2 summarizes the clinical studies that link Maqui with cardiovascular protection. These results show the effect of different extracts of this berry on cardiovascular risk factors (glucose index, obesity, diabetes, among others). Some studies showed the biological activity of the Maqui extract, while others relate the biological potential with the presence of some compounds of a mainly phenolic nature.

## 5. Antiplatelet Activity of the Compounds in Maqui

Several fruits such as red grapes, strawberries, kiwis, and pineapples have been shown to exert antiplatelet effects [63]. The most commonly investigated berries with antiplatelet potential have been grapes, aronia berries, and sea buckthorn berries, which contain phenolic compounds such as hippuric acid, pyrogallol, catechol, and resorcinol which inhibit collagen-stimulated platelets at a concentration of 100 µM [83,84]. A combination of extracts from different berries (blueberries, strawberry puree, cranberries, blackcurrant puree, and raspberry juice) showed an antiplatelet effect when using ADP and collagen as agonists, in platelets from healthy adults who consumed moderate amounts of berries for 8 weeks [84,85]. Another in vivo study, in whole blood, showed that the combination of grape seed and skin extracts reduced collagen-induced platelet aggregation to a greater extent than either extract alone, showing the importance of synergistic inhibitory actions at physiological levels [84,86]. Cranberry juice also inhibited ADP- and collagen-induced platelet aggregation after four days of consumption four times a day [87]. In addition, strawberry extract (0.1–1 mg/mL) in an in vivo model inhibits platelet aggregation induced by arachidonic acid (AA) and ADP in a dose-dependent manner [44]. Results of in vitro and ex vivo studies have reported that polyphenols present in red grape and purple grape juices inhibit platelet aggregation induced by ADP, thrombin, collagen, epinephrine, and AA [85,88,89,90,91]. Chlorogenic acid, a polyphenol present in cherries, apples, kiwis, eggplants, plums, and coffee, also exhibits antiplatelet activity [92]. 

The few studies about antiplatelet activity in Maqui are recent [93]. The aggregation and platelet secretion induced by ADP and collagen were significantly inhibited by leaf and immature fruit extracts of the varieties “Luna Nueva” and “Morena”. These extracts also reduced oxidative stress, an effect that might be related to the high content of antioxidant compounds [20]. The chemical characterization allowed us to identify several compounds with known antiplatelet potential, such as caffeic acid, quercetin, isorhamnetin, kaempferol, and rutin, among others. The varieties differ in the composition and concentration of phenolic compounds, supporting the fact that extracts from different genotypes and parts of the tree, e.g., immature compared to mature fruit, vary in their antiplatelet activities. The participation of each bioactive compound with antiplatelet activity was also investigated using Pearson’s statistical analysis. The results showed that the levels of phenolic compounds are responsible for the antiplatelet effects of Maqui [93].

Although this berry has been attributed with a wide biological potential, a gap can be seen in the literature regarding its antiplatelet properties. However, its rich chemical composition clearly shows that the presence of chemical compounds with high antiplatelet potential can give Maqui a prominent potential in the development of new antiplatelet agents. Table 3 summarizes some in vitro and in vivo studies of the antiplatelet activity of bioactive compounds identified in Maqui, i.e., anthocyanins, flavonoids, and phenolic acids that have important antiaggregant activity against several agonists, e.g., TRAP-6, ADP, collagen, and arachidonic acid (AA). 

### 5.1. Anthocyanins

The anthocyanins delphinidin-3-*O*-rutinoside, cyanidin-3-*O*-glucoside, cyanidin-3-*O*-rutinoside, and malvidin-3-*O*-glucoside inhibit platelet aggregation [30,31,96,98,111,118] using TRAP-6, epinephrine, collagen, and ADP as agonists [30,96,98,101,109,110,111,116,118]. The effects were even greater in mixtures of these compounds, possibly due to the synergism of polyphenols and anthocyanins.

The use of pure chemical compounds in studies on platelet function is vital to elucidate the mechanism of antiplatelet action. Yan et al. determined the effects of delphinidin-3-*O*-glucoside on platelet activation in both in vitro and in vivo models of thrombosis, concluding a dose-dependent antiplatelet effect in human platelet-rich plasma (PRP) and washed platelets, activated with collagen, ADP, and TRAP-6 [31]. Delphinidin-3-*O*-glucoside also inhibited the growth of the thrombus in mice and the exposure of P-selectin in the platelet membrane. Furthermore, the authors reported that this compound inhibited the phosphorylation of the adenosine-activated protein kinase (AMPK) [31]. The concentrations of anthocyanins used in the cited experiments were within the physiological levels and the pharmacological dose (0.5–50 µM) [30]. 

In the presence of anthocyanins, thrombus formation in mice and human in vivo perfusion chambers was reduced at low and high shear rates [31]. In a model of intravital microscopy thrombosis, anthocyanins prolonged the time for thrombus formation at a dose of 0.5 µM and delayed vessel occlusion significantly at 5 µM and 50 µM. The release of α and dense granules, the expression of P-selectin, the cluster of differentiation (CD) 63 (CD63), CD40L, and the secretion of the cytosolic proteins were observed. In addition, delphinidin-3-*O*-glucoside decreased the expression of the α_2_bβ_3_ integrin. Overall, the cited study suggests that the daily consumption of anthocyanins may play a fundamental role in the protection against CVD, which could be related to the inhibition of platelet activity [31].

### 5.2. Flavonols

Quercetin is one of the main flavonoids identified in several natural sources, including Maqui [119]. Both antiplatelet potential and other biological properties have been attributed to this flavonol. Quercetin inhibits platelet aggregation induced by collagen, with an IC_50_ = 6 μg/mL at a concentration of 5 μg/mL of collagen [120]. The antiplatelet mechanism of this flavonoid has been related to the inhibition of collagen-stimulated tyrosine phosphorylation, a key component of the collagen signaling pathway through glycoprotein VI, Syk [121]. This compound has not only shown potent activity against collagen; previous studies report that it inhibits platelet aggregation induced by AA (substrate of cyclooxygenase-1 (COX-1)) and by U46619 (synthetic mimetic of TxA_2_) [122].

Previous works show the cardioprotective potential of quercetin and kaempferol, as well as their derivatives. Quercetin 3-*O*-[(6-*O*-E-feruloyl)-β-D-glucopyranosyl-(1→2)]-β-D-galactopyranoside-7-*O*-β-D-glucuropyranoside) and kaempferol 3-*O*-[(6-*O*-E-caffeoyl)-β-D-glucopyranosyl-(1→2)]-β-D-galactopyranoside-7-*O*-(2-*O*-E-caffeoyl′)-β-D–glucuropyranoside isolated from *Lens culinaris* Medik. showed potent antiplatelet action. Results revealed decreased collagen adhesion of resting platelets and thrombin-activated platelets after incubation with quercetin and kaempferol derivatives [105]. On the other hand, kaempferol has shown its potential to reduce and prevent thrombosis. The background shows that this flavonoid inhibits fibrin polymer formation, attenuates phosphorylation of extracellular signal-regulated kinase (ERK)1/2, p38, c-Jun N-terminal kinase (JNK)1/2, and phosphoinositide 3-kinase (PI3K)/PKB (AKT) in thrombin-stimulated cells and decreases collagen/epinephrine-stimulated platelet aggregation by 34.6%. Additionally, kaempferol protected mice from thrombosis in models of acute thromboembolism induced by collagen/epinephrine and thrombin, as well as carotid artery thrombus induced by FeCl_3_ [104]. Another flavonol that has been identified in Maqui and that stands out for its antiplatelet action is myricetin. This compound reduces the ability of platelets to spread over collagen and form thrombi in vitro. This effect has been attributed mainly to the inhibition of PDI and ERp5 by binding to myricetin, forming non-covalent bonds [107]. Additionally, myricetin has been shown at physiologically relevant concentrations to inhibit platelet aggregation induced by TRAP-6 and AA. It also inhibits fibrinogen binding and collagen-related peptide-induced alpha granule secretion [123]. The inhibitory effect against several platelet agonists suggests that this flavonoid can act on molecules common to different pathways [107,123].

Isorhamnetin was one of the compounds that presented a positive correlation between its concentration and the antiplatelet activity of Maqui extracts [93]. The effect of isorhamnetin on mitochondrial function, platelet adhesion, and thrombus formation was evaluated under conditions of controlled blood flow in the Badimon perfusion chamber. This flavonol showed antiplatelet activity induced by collagen, and thrombin receptor activator peptide-6 (TRAP-6), with IC_50_ values of 8.1 ± 2.6 µM and 16.1 ± 11.1 µM, respectively [124]. It also decreased the mitochondrial membrane potential and reduced platelet deposition on the thrombus, confirming its antithrombotic effect [124].

Isorhamnetin and tamarixetine, quercetin methylated metabolites, stand out for their antiplatelet potential, even showing better results than aspirin in an aggregometry test. These compounds inhibit human platelet aggregation and suppress activating processes, including granule secretion, αIIbβ3 integrin function, calcium mobilization, and spleen tyrosine kinase (Syk). They also attenuated thrombus formation in an in vitro microfluidic model, while isorhamnetin inhibited thrombosis in a mouse model of laser injury [125]. 

### 5.3. Flavones

Eriodictyol, a flavone present in Maqui, inhibits platelet aggregation stimulated by collagen and AA, in platelet-rich plasma (IC50 = 912.9 ± 37 μM and IC50 = 1027.3 ± 551 μM, respectively) [110]. On the other hand, hesperetin can selectively inhibit collagen- and AA-mediated signal transduction, with IC50 of 20.5 ± 3.5 and 69.2 ± 5.1 μM, respectively. The proposed mechanism suggests the inhibition of PLC-γ2 phosphorylation and the activity of COX-1 [111].

### 5.4. Phenolic Acids

Caffeic acid, identified in Maqui, has also been noted for its antiplatelet properties. Studies have shown that this compound possesses antithrombotic activity on mouse brain arterioles in vivo, and inhibits platelet aggregation in vitro stimulated by various agonists (ADP and thrombin) [126,127]. It has been described that this polyphenol is a potent compound that increases the level of cAMP-dependent protein phosphorylation in collagen–platelet interactions [128]. Studies report its antithrombotic action at doses of 1.25–5 mg/kg, an effect related to its capacity to suppress phosphorylation of ERK, p38, and JNK, which leads to cAMP elevation, and it negatively regulates P-selectin expression and activation of αIIbβ3 [126,127,128]. Meanwhile, Nam et al. showed that this polyphenol decreases the production and release of thrombogenic molecules in human platelets. This effect was not only mediated by TxA_2_ but also by the decrease in serotonin released by collagen by inhibiting the phosphorylation of JNK1 [129]. This phenolic compound at 25–100 μM further inhibited ADP-induced platelet aggregation, P-selectin expression, ATP release, Ca^2+^ mobilization, and αIIbβ3 integrin activation [127]. 

The potential of ferulic acid has been related to the activation of cAMP and cGMP signaling [113,114]. This compound inhibits dose-dependent (50–200 µM) platelet aggregation induced by platelet agonists (ADP, thrombin, collagen, AA, and U46619). Additionally, it attenuates intracellular Ca^2+^ mobilization and TxA2 production. It also increases cAMP, cGMP, and vasodilator-stimulated phosphoprotein (VASP) levels while decreasing phospho-MAPK and phosphodiesterase (PDE) in washed rat platelets [113]. Studies report that ferulic acid has an antithrombotic effect in the in vivo model of acute thromboembolism and decreases the expression of αIIbβ3/FIB and AKT phosphorylation in thrombin-stimulated platelet activation [114].

Both ellagic acid and ferulic acid inhibit platelet activation in vitro, induced by ADP and collagen [130]. Phenolic acid derivatives have also been shown to be novel antiplatelet targets, even more potent than their phenolic precursors. Dihydrocaffeic acid and dihydroferulic acid at doses of 0.01–100 μg/mL 1 μM decreased ADP-stimulated platelet activation, measured as P-selectin expression and fibrinogen binding [131].

AA, arachidonic acid; ADP, adenosine diphosphate; ATP, adenosine triphosphate; Akt, protein kinase B; CD63, a membrane protein associated with lysosome 3; CD40L, a ligand of the membrane protein; COX-1, cyclooxygenase; CRP, collagen-related peptide; ERK: extracellular signal-regulated kinase; FIB, plasma fibrinogen; FXa, factor Xa; cGMP, cyclic guanosine monophosphate; GP, glycoprotein; 5-HT, serotonin; αllbβ3, GPVI, glycoprotein VI; GPIIb/IIIa, glycoprotein IIb–IIIa; JNK: c-Jun N-terminal kinase, MAPK, protein kinase activated by mitogens; MW, molecular weight; OH*, hydroxyl radical; PAF, platelet activating factor; PDE: phosphodiesterase; PG, glycoprotein; PF4, platelet factor 4; PI3, phosphoinositol 3 kinase; PKB, protein kinase B; PKC, protein kinase C; PLC γ2, phospholipase C; Rantes, regulatory chemokine beta; ROS, reactive oxygen species; Ser, serotonin; Syk, tyrosine protein kinase; β-TG, beta thromboglobulin; TGF-β1, transforming growth factor-beta 1; TRAP-6, thrombin receptor activator for peptide 6; TxA_2_, thromboxane; VASP, phosphoprotein stimulated by vasodilator. 

The secretion of α and dense granules, the release of adenosine triphosphate (ATP), and the mobilization of Ca^2+^ are the main markers of activation reported to date by mechanisms involving the AA-derived pathway and GPVI receptor, among others. Some receptors for collagen (GPVI), thrombin (protein activated receptors, PARs), and TxA_2_ activate phospholipase C (PLC). The aforementioned receptors generate diacylglycerol (DAG) and inositol triphosphate (IP3), activating the protein kinase C (PKC) and intracellular Ca^2+^ release, leading to granule secretion [98,101,102]. On the other hand, adenylyl cyclase (AC) favors the conversion of ATP into cyclic adenosine monophosphate (cAMP). The activation of phosphoinositide 3-kinase/protein kinase B (PI3K/Akt) could mediate the phosphorylation of endothelial nitric oxide synthase (eNOS), increasing the production of platelet nitric oxide (NO). This leads to the production of cyclic guanosine monophosphate (cGMP), and stimulates the activation of mitogen-activated protein kinases (MAPK), thus promoting granule secretion and activation of platelets [31,118]. 

**Figure 5:**
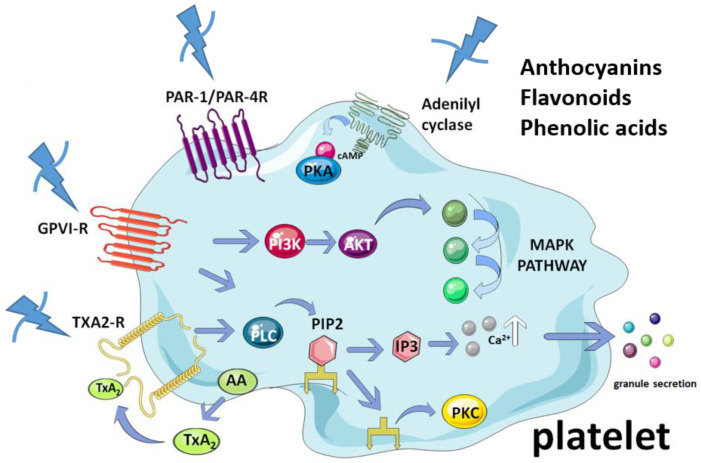
Antiplatelet mechanistic proposal for Maqui. AC, adenylate cyclase; AKT, protein kinase B; cAMP, cyclic adenosine monophosphate; DAG, diacylglycerol; PI_3_, phosphoinositol 3 kinase; PIP2, phosphatidylinositol bisphosphate; GP, glycoprotein; MAP, mitogen-activated protein kinase; P2Y12/P2P1, ADP receptor; TxA_2_R, thromboxane receptor.

Considering the studies about the antiplatelet activity of previous tested chemical compounds that have also been identified in Maqui, and the reviewed inhibition mechanisms of platelet activation, we propose a scheme of how the bioactive compounds present in Maqui extracts exert their effects (Figure 5).

## 6. Limitations and Future Perspective 

Inhibition of platelet function has long been used to prevent and treat CVD [63]. Although antiplatelet drugs currently exist, this therapy has been accompanied by side effects, such as bleeding. Recent efforts focus on the search for and development of new therapeutic agents, together with the healthy habits that we must promote to contribute to adequate cardiovascular health.

In general, healthy eating is promoted, which includes minimally processed foods and foods rich in bioactive products such as fruits, nuts, seeds, beans, vegetables, whole grains, vegetable oils, yogurt, and fish [132]. Epidemiological studies have provided evidence for the protective role of healthy diets in the prevention of CVD. For example, eating two or three kiwis a day for 28 days reduces platelet aggregation induced by collagen and ADP. On the other hand, raw garlic and some of its preparations are recognized as antiplatelet agents [63]. As the above, Maqui plays a fundamental role among healthy fruits due to its wide biological potential, as highlighted in this work.

Maqui is known to be rich in phenolic compounds, and phenolic compounds have a well-recognized role in reducing the risk of chronic diseases, increasing healthy life years, and promoting healthy aging [133]. The protective mechanisms of foods rich in polyphenols may not only depend on their content of nutrients and bioactive compounds but also include their food matrix properties that affect glycemic load and energy density, among others [132]. 

The extraction procedure affects the chemical composition of the Maqui extracts; variables such as temperature, sonication, extraction time, and solvent are some of the parameters that would influence the chemical profile identified for this species [119]. Concerning the solvent used for extractions in Maqui matrices, it has been shown that hydroalcoholic mixtures are the most used solvents for the extraction of bioactive components from berries [134]. The biological potential of aqueous and ethanolic extracts of Maqui, which have a high content of polyphenols, has been reported [28,46]. Hydroalcoholic extract presented higher polyphenolic content, with antioxidant and antimicrobial properties meaning it is the most promising extract for pharmaceutical purposes [51]. As previously described, Maqui has the highest phenol content compared to other berries, and this depends substantially on the solvent used for extraction [16,19].

Sonication is an ideal alternative to obtain extracts from Maqui berries with high bioactivity [49] while the extraction time can also modify the antioxidant capacity of Chilean Maqui berries [48]. Three operating conditions of the extraction process were compared by modifying the type of solvent (methanol, ethanol, and acetone), solvent concentration (20, 60, and 100%), and extraction time (15, 127.5, and 240 min). Results show that after a certain extraction time there is a final equilibrium between the solutes of the matrix and the extraction solvent [28]. 

Metabolites such as polyphenols, flavonoids, and anthocyanins are the main ones that have been identified in Maqui extracts. Polyphenols may possess antiplatelet properties, but their coadministration may not be safe [135]. It is important to make the correct and precise determination of both the composition and the amounts of the phenolic compounds that we consume to avoid toxicity or unwanted side effects. Of the total number of trials with polyphenols in the last 20 years, 20% analyzed vascular and endothelial responses, and trials on platelet function and thrombosis are lacking [135,136]. In vivo and trial studies evaluating potential polyphenol–drug interactions are needed to address this limitation [123].

On the other hand, studies refer to the protective effects of flavonoids against drug-induced toxicity. It was concluded that flavonoids, both dietary and derived from plant medicines, can exert protective effects against drug-induced toxicity [135,137,138]. These compounds are generally recognized as safe, due to the long history of use and consumption of foods rich in flavonoids. The total daily intake of flavonoids in Europe is estimated to be around 428 ± 49 mg, with 136 ± 14 mg being monomeric [135,139], while flavonoid-rich beverages and vegetables can reach 1000 mg/day of flavonoids [135,140].

Although the benefits of phenols have been demonstrated in different in vitro and in vivo models, there are few reports evaluating the possible effects of Maqui extracts on platelet aggregation [93]. Although there are clinical studies that show that Maqui extract or capsules are safe for human consumption [74,75], studies that evaluate their administration over longer periods are required.

Although in vitro studies help to understand the possible health contributions of Maqui berries, these may not be fully applicable to humans, as in the case of anthocyanins, such as delphinidin and anthocyanin, which have been shown to have limited bioavailability. In animal and human studies, anthocyanins are poorly absorbed and thus show low bioavailability [73]. Furthermore, several groups have suggested that dietary cyanidins and delphinidins may be subject to extensive biotransformation in humans, most likely involving the colonic microbiota [73,141].

Despite the above, it appears that anthocyanins are more bioavailable than is perceived, and their metabolites are present in the circulation for ≤48 h after ingestion. A study carried out on eight male volunteers showed that cyanidin-3-*O*-glucoside, a main component of the Maqui berry, has a non-negligible bioavailability of 12.38 ± 1.38%. Blood concentrations of cyanidin-3-*O*-glucoside and cyanidin-3-*O*-glucoside conjugates were observed to appear between 1 and 2 h after the intake of 500 mg of cyanidin-3-*O*-glucoside [142].

There are few in vivo and in vitro studies comparing Maqui with other fruits and berries to determine the benefits and weaknesses of Maqui compared to other foods with similar bioactive compound composition. There is a lack of studies to establish how much Maqui should be consumed daily to see this effect at physiologically relevant concentrations.

The natural bioactive ingredients of Maqui have antiplatelet effects with multiple targets on the platelet, while the synergistic effects of the phenolic compounds could enhance the antiplatelet activity. Due to the above, there exists an interest in the cardiovascular benefits of Maqui berries, so daily consumption of this berry can prevent the development of CVD, although detailed studies are needed to reinforce its clinical utility.

## 7. Conclusions

Due to the prevalence of CVD, new antiplatelet drugs are needed to prevent and treat arterial thrombosis as well as other CVDs. Some bioactive compounds, such as polyphenols and anthocyanins in fruits and vegetables, have been reported to inhibit platelet activation and so reduce the risk of CVD.

The broad chemical profile of Maqui (flavonoids, anthocyanins, and phenolic acids) is directly related to its high biological potential. The current knowledge about Maqui’s antioxidant, anti-inflammatory, and hypoglycemic effects suggests that a diet including Maqui could aid in the prevention of CVD, with more studies being required to prove this hypothesis. The fundamental mechanisms which this species influences have been mainly related to the inhibition of lipid peroxidation, decrease in cholesterol and blood glucose levels, as well as a decrease in oxidative stress. Studies are needed to establish how much Maqui should be consumed daily to see this effect at physiologically relevant concentrations.

Additionally, this species can be studied in detail for antiplatelet purposes, since to date, there have been few authors who have highlighted this potential in the fruits of Maqui. Our findings highlighted that the main mechanism by which the compounds identified in this species act is related to the metabolic pathways of the AA and GPVI receptors. Although anthocyanins are the main phenolic compounds in this berry, antiplatelet activity may be directly related to the presence of a specific compound or enhanced by the synergy of several phenolic compounds.

This review allowed us to investigate the antiplatelet and cardioprotective activity of the bioactive compounds present in Maqui and highlight the areas in which much remains to be investigated. Without a doubt, we have shown that Maqui is an interesting target in the search for new antiplatelet therapies.

## Figures and Tables

**Table 2:** Cardioprotective role of Maqui: clinical studies.

Plant Part(s)	Type of Extract	Dose or Concentration	Mechanism of Action or Effect of Extract and/or Pure Compound Characterization of the Extract	Characterization	Identified Compound	Reference
Fruit	Methanolic extract of ripe fruits of Maqui.	100, 10, and 1 ppm/kg of rat body weight	**Cardioprotection** Methanolic extract of ripe fruits of Maqui has antioxidant activity and cardioprotective effect on acute ischemia/reperfusion performed in rat hearts. The extract protected from heart damage due to the incidence of reperfusion dysrhythmias and non-recovery of sinus rhythm. It also prevents harmful events in the heart of the animal by reducing lipid oxidation and reducing the concentration of substances reactive to thiobarbituric acid, and lipid peroxidation index.	No		[20]
Fruit	Maqui berry extract	150 mg standardized	**Oxidative stress biomarkers** Delphinol reduces levels of Ox-LDL and urinary F2-isoprostanes (8-iso-prostaglandin F2α).	No		[81]
Fruit	Maqui berry powder (ground whole fruit rich in anthocyanins)	50 and 100 mg/kg	**Oxidative stress markers** The administration of the aqueous extract of Maqui berry prevents the cognitive deficit caused by chronic exposure to ozone. Decreases levels of oxidative stress markers and superoxide enzymatic activity in animals exposed to ozone through four oxidative stress markers: 4HNE, MDA, Nt3, and AGEs in brain areas involved in learning and memory processes.	Yes	Ellagic acid derivatives, flavonols, and chlorogenic acid.	[82]
Fruit	Hydroethanolic extract of a Chilean berry Maqui. Pure compounds: rutin and quercetin	500 µg/mL (extract) 50 µM (quercetin) and 10 µM (rutin)	**Endothelial dysfunction and oxidative stress** Maqui berry extracts, quercetin, and rutin protect against endothelial dysfunction induced by high glucose and pyrogallol through increased generation and bioavailability of NO.	Yes	p-Coumaric acid, rutin, gentisic acid, sinapic acid, procyanidin B; gallic acid, quercetin, myricetin, delphinidin-3-*O*-glucoside, cyanidin-3-*O*-glucoside, delphinidin-3,5-*O*-diglucoside, delphinidin-3-*O*-sambubioside, cyanidin-3-*O*-sambubioside, proanthocyanidin B, proanthocyanidin blend, catechin epicatechin blend, p-coumaric acid and p-hydroxybenzoic acid blend, cyanidin catechin blend and free sugar blend.	[72]
Fruit	Hydroalcoholic extract of Maqui. Pure compound: rutin	50 mg (extract) 30 mg/kg (rutin)	**Vascular reactivity, hyperglycemia, and dyslipidemia** Maqui reduces plasma levels of cholesterol, LDL and triglycerides. Rutin lowers blood sugar and enhances endothelium-dependent relaxation. Maqui and rutin improved the bioavailability of nitric oxide.	Yes	Gentisic acid, ferulic acid, gallic acid, p-coumaric acid, sinapic acid, 4-hydroxybenzoic acid, delphinidin, cyanidin, vanillic acid, quercetin, myricetin, mixed catechin and epicatechin, delphinidin, delphinidin-3-*O*-sambubioside-5-*O*-glucoside, delphinidin- 3,5-*O*-diglucoside, cyanidin-3-*O*-sambubioside-5-*O*-glucoside, cyanidin-3,5-*O*-diglucoside, delphinidin-3-*O*-sambubioside, delphinidin-3-*O*-glucoside, cyanidin-3-*O*-sambubioside, and proanthocyanidin B.	[71]
Fruit	Maqui extract enriched with anthocyanins. Pure compound: delphinidin-3-*O*-sambubioside-5-*O*-glucoside	125–500 mg/kg (extract) 2–10 μg/mL and 5–100 μg/mL (delphinidin-3-*O*-sambubioside-5-*O*-glucoside)	**Type 2 diabetes** Oral administration of anthocyanins reduces fasting blood glucose levels and glucose tolerance in hyperglycemic obese C57BL/6J mice fed a high-fat diet. Oral administration of delphinidin-3-*O*-sambubioside-5-*O*-glucoside dose-dependently lowered fasting blood glucose levels in obese C57BL/6J mice (2–10 μg/mL). It also decreases glucose production in rat liver cells (50–100 μg/mL).	Yes	Delphinidin-3-*O*-sambubioside-5-*O*-glucoside, delphinidin-3,5-*O*-diglucoside, delphinidin3-*O*-sambubioside, delphinidin-3-*O*-glucoside, cyanidin-3-*O*-sambubioside, cyanidin-3-*O*-glucoside, cyanidin-3-*O*-sambubioside-5-*O*-glucoside + cyanidin-3,5-*O*-diglucoside.	[75]
Fruit	Standardized extract of berries of Maqui. Pure compound: delphinidin	20 mg/kg (extract) 50 µM (delphinidin)	**Postprandial blood glucose** Delphinol^®^ lowers blood glucose and postprandial insulin. Daily oral application of Delphinol^®^ for four months reduces fasting blood glucose levels and lowers postprandial glycemia due to sodium–glucose cotransporter inhibition in the small intestine.	No		[73]
Fruit	Maqui	250 mL containing approx. 1000 μmol GAE of polyphenols)	**Postprandial blood glucose** Reduction in the glycemic peak mediated by the glucose + Maqui + lemon mixture. Mixing glucose + Maqui + lemon reduces the glycemic peak of glucose (20.5 ± 8.4%) compared to glucose. This amount represents a reduction of 36.7 ± 15.0 mg/dL in postprandial blood glucose.	No		[75]
Fruit	Maqui extract	20 mg of freeze-dried Maqui/mL of filtered tap water	**Obesity** Maqui extract prevents diet-induced obesity and its associated comorbidities. Reduced fasting glucose. Improves insulin response and reduces weight gain, and also a differential expression of genes involved in de novo lipogenesis.	Yes	Delphinidin-3-*O*-sambubioside-5-*O*-glucoside, delphinidin-3-*O*-sambubioside, cyanidin-3-*O*-sambubioside-5-*O*-glucoside, cyanidin-3-*O*-glucoside, cyanidin-3-*O*- sambubioside.	[74]

**Table 3:** Antiplatelet activity of anthocyanins and phenolic compounds in Maqui.

Compounds	In Vitro	In Vivo	Reference
**Anthocyanins**			
Delphinidin-3-*O*-glucoside (5–50 µg/mL)	Inhibition of platelet aggregation with collagen (10 μg/mL) and TRAP-6 (100 μM) at 0.5 μM and 50 μM in washed platelets. Inhibition of platelet aggregation with ADP (5 μM), collagen (10 μg/mL) and TRAP-6 (100 μM) at 0.5 μM and 50 μM in platelet-rich plasma Dose-dependent reduction in activated GPIIb/IIIa expression. Inhibition of platelet adhesion and aggregation in perfusion chamber assays at low and high shear rates. Decreased platelet deposition, thrombus formation, and vessel occlusion.		[94]
	Inhibition of platelet aggregation with ADP (5 μM), collagen (2 μg/mL), and TRAP (100 μM). Inhibition of the activation and secretion of P-selectin, CD63, CD40L, αllbβ3, and fibrinogen with ADP (200 μM), collagen (10 μg mL), thrombin (1 U/mL), and TRAP (250 μM). Mechanism: inhibition of the phosphorylation of MAPK induced by collagen (25 μg/mL).	Inhibition of collagen-induced thrombus formation (100 μg/mL), using controlled flow. Inhibition of thrombus formation induced by FeCl_3_ at 50 μg/mL, using intravital microscopy.	[31,95]
Cyanidin-3-*O*-glucoside (5–50 µg/mL)	Inhibition of the activation and secretion of P-selectin, CD63, CD40L, αllbβ3, fibrinogen with collagen (10 µg/mL), thrombin (2 U/mL), and TRAP (250 µM). Inhibition of platelet aggregation with collagen (2.5 µg/mL), thrombin (0.1 U/mL), and TRAP (100 µM). Mechanism: via receiver GPVI collagen (2.5 µg/mL). Inhibition of the phosphorylation of tyrosine protein induced by collagen (2.5 µg/mL) at 5–50 µM.	Inhibition of the formation of the thrombus induced by collagen (0.5–50 µM) and FeCl_3_ at 5–50 µM.	[95,96]
	Inhibition of platelet aggregation with collagen (10 μg/mL) and TRAP-6 (100 μM) at 0.5 μM-and 50 μM in washed platelets. Inhibition of platelet aggregation with ADP (5 μM), collagen (10 μg/mL) and TRAP-6 (100 μM) at 0.5 μM and 50 μM in platelet-rich plasma Dose-dependent reduction in activated GPIIb/IIIa expression. Inhibition of platelet adhesion and aggregation in perfusion chamber assays at low and high shear rates. Decreased platelet deposition, thrombus formation, and vessel occlusion.		[94]
	Inhibition of platelet granules (P-selectin, CD40L, 5-HT, RANTES, and TGF-β1) with thrombin (0.5 U/mL).	Attenuated serum levels of PF4 and β-TG in mice fed high-fat diets at a dose of 1000 mg/kg.	[97]
**Flavonols**			
Quercetin- 4”-*O*-β-D-glucoside		Inhibition of platelet aggregation with collagen (50 μL) at 150 mg. Mechanism: inhibition of the phosphorylation of protein tyrosine kinase Syk and PLCγ2 induced by collagen (25 μg/mL) at 150 mg.	[98]
Quercetin	Inhibition of platelet aggregation with AA (100 μM), ADP (20 μM), collagen (10 μg/mL) at 13 μM. It inhibits ATP release with ADP (7 μM) and epinephrine (7 μM) at 2.5 μM. Mechanism: inhibits the formation of TxA_2_ and PG induced by AA (100 μM) at 5 μM.		[99]
	Inhibition of platelet aggregation with 100 μg/mL of AA (100 μM) and collagen (10 μg/mL) at 100 μg/mL.	Relaxation in the thoracic aorta of the rat is induced by norepinephrine (3 μM) at 100 μM.	[100]
	Inhibition of platelet aggregation with collagen (0.5–5 μL/mL) at IC_50_: 2.37–8.69. Inhibition of the mobilization of Ca^2+^ induced by collagen (5 μL/mL) at 15 μM. Mechanism: inhibits the GPVI signaling pathways, phosphorylation of tyrosine protein, and PI3 kinase induced by collagen (25 μL/mL) at 25 μM.		[101]
	Inhibition of platelet aggregation with collagen (0.5–5 μL/mL) at IC_50_: 2.37–8.69. Inhibition of the mobilization of Ca^2+^ induced by collagen (5 μL/mL) at 15 μM. Mechanism: inhibits the GPVI signaling pathways, phosphorylation of tyrosine protein, and PI3 kinase induced by collagen (25 μL/mL) at 25 μM.		[102]
	Inhibition of platelet aggregation with AA (150 μM) IC_50_: 18 μM. The increase in cAMP stimulated by PGI_2_ (0.5 nM) decreased at 50 μM. Mechanism: inhibition of the activity of COX-1 and lipoxygenase at 10 μM and 50 μM.		[103]
Kaempferol	Inhibition of thrombin (40 mU) and FXa (20 mU) (68 ± 1.6% and 52 ± 2.4%, respectively). Attenuated fibrin polymer formation in turbidity and phosphorylation of ERK 1/2, p38, JNK 1/2, and phosphoinositide PI3K/PKB (AKT) in cells stimulated with thrombin (0.5 U/mL). Inhibition of platelet aggregation stimulated by collagen/epinephrine (34.6%). Mechanism: inhibition of phosphorylation of ERK 1/2, p38, JNK 1/2, and PI3K/PKB.	Decreased thrombus formation in 3 animal models (collagen/epinephrine and thrombin-induced acute thromboembolism, FeCl_3_-induced model, and carotid arterial thrombus model).	[104]
	Decreased collagen adhesion in resting platelets and activated platelets with thrombin at a dose of 5 μg/mL. Inhibition of platelets activated by thrombin and fibrinogen (40%). Inhibition of platelet aggregation with collagen (5 μg/mL) and AA (0.5 μmol/L) at 50 μg/kg. Thrombin-stimulated reduction of enzymatic lipid peroxidation in platelets.		[105]
Myricetin	Inhibition of platelet aggregation with collagen (5 μg/mL) and AA (0.5 μmol/L) at 50 μg/kg. Thrombin-stimulated reduction in enzymatic lipid peroxidation in platelets.		[106]
	Inhibition of platelet aggregation and secretion of alpha granules. with TRAP-6 (10 µM) and collagen (1 µg/mL) at 15 and 30 µM. Decreased fibrinogen binding induced by CRP (1 µg/mL) and TRAP-6 (10 µM) at 15 μM. Reduction in adhesion on collagen and thrombus formation without affecting hemostasis in vivo. Mechanism: inhibition of ERp5 and PDI.		[107]
	Dose-dependent (20–30 µM) inhibition of platelet aggregation, granule secretion and activation (activation of αIIbβ3 integrin and P-selectin exposure), generation of ROS, and induced intracellular Ca^2+^ mobilization by CRP (0.1 µg/mL) and collagen (1 µg/mL). Mechanism: inhibition of GPVI during cell activation.	Reduction in ischemia/reperfusion-induced acute infarction in a mouse model of stroke. Blocked FeCl_3_-induced arterial thrombus formation in vivo and thrombus formation on collagen-coated surfaces under low shear rate.	[108]
Rutin	Inhibition of platelet aggregation with collagen at 250 μM (1 μg/mL). The mobilization of Ca^2+^ induced by collagen (1 μg/mL) decreases to 250 μM. Mechanism: inhibition of the PLC phosphorylation and formation of TxA2, inhibits collagen-induced phosphorylation of P47 at 250 μM.		[109]
**Flavanones**			
Eriodictyol	Inhibition of platelet aggregation with collagen (2 μg/mL) and AA (0.5 mmol/L) at 50 μM.		[110]
Hesperetin	Concentration-dependent inhibition of platelet aggregation induced by collagen (5 μg/mL) and AA (0.5 μmol/L) (IC_50_: 20.5 and at IC_50_: 69.2, respectively). Inhibition mobilization of cytosolic Ca^2+^ induced by collagen (10 μg/mL) at 20–50 μM. Inhibition of the secretion of serotonin with collagen (5 μg/mL) and AA (0.5 μmol/L) at IC_50_: 10.5 and at IC_50_: 25.2, respectively. Mechanism: inhibitionPLC-γ2 phosphorylation. Inhibition of COX-1 activity.		[111]
		Atherosclerosis inhibition	[112]
**Phenolic acids**			
Ferulic acid	Inhibition of platelet aggregation induced by ADP, thrombin (0.5 U/mL), AA (2 mM), collagen (2 μg/mL), and U46619 (2 μM) at 50–200 µM. Inhibition of mobilization of cytosolic Ca^2+^ and TXB_2_ production. Increased the levels of cAMP and cGMP and phosphorylated VASP. Decreased phospho-MAPK and PDE. Mechanism: activation of cAMP and cGMP signaling.	Decreased pulmonary thrombosis and prolonged tail bleeding and coagulation time in mice without altering coagulation parameters.	[113]
	Inhibition of platelet activation (serotonin secretion) stimulated by thrombin, collagen/epinephrine, and decreased clot retraction activity at 10 μg. Mechanism: decreased granule secretion, prolongation of the intrinsic coagulation cascade, and upregulation of αIIbβ3/FIB/AKT signaling expressions.	Decreased thrombosis in acute thromboembolism model and decreased αIIbβ3/ FIB expression and AKT phosphorylation in thrombin-stimulated platelet activation.	[114]
Caffeic acid	Inhibition of platelet aggregation with ADP (8 μmol/L) and collagen (1.5 μg/mL) at 0.5 mmol/L.		[115]
	Inhibition of the activation and secretion of P-selectin with TRAP (25 μmol/L) at 100 μmol/L.		[83]
	Inhibition of platelet aggregation with collagen (2 μg/mL) at 15–25 μM. Mechanism: inhibition of the phosphorylation of cGMP/VAS Ser /VASP Ser157 at 15–25 μM. Decreases PKC and phosphorylation of P47 at 15–25 μM.		[116]
Ellagic acid	Inhibition of platelet aggregation with collagen (1 μg/mL) at IC_50_: 50 μM. The mobilization of Ca^2+^ induced by collagen (1 μg/mL) decreases at 50 μM. Mechanism: inhibition of the PLCγ2-PKC cascade, OH* formation, MAPKs, and Akt induced by collagen (1 μg/mL) at 50 μM.		[117]
